# Moving in extreme environments: what’s extreme and who decides?

**DOI:** 10.1186/2046-7648-3-11

**Published:** 2014-06-11

**Authors:** James David Cotter, Michael J Tipton

**Affiliations:** 1 School of Physical Education, Sport and Exercise Sciences, Division of Sciences, University of Otago, PO Box 56, Dunedin 9054, New Zealand; 2 Extreme Environments Laboratory, Department of Sport and Exercise Science, University of Portsmouth, Portsmouth PO1 2ER, UK

**Keywords:** Stress, Autonomy, Regulation, Injury, Illness, Adaptation, Guidelines

## Abstract

Humans work, rest and play in immensely varied extreme environments. The term ‘extreme’ typically refers to insufficiency or excess of one or more stressors, such as thermal energy or gravity. Individuals’ behavioural and physiological capacity to endure and enjoy such environments varies immensely. Adverse effects of acute exposure to these environments are readily identifiable (e.g. heat stroke or bone fracture), whereas adverse effects of chronic exposure (e.g. stress fractures or osteoporosis) may be as important but much less discernable. Modern societies have increasingly sought to protect people from such stressors and, in that way, minimise their adverse effects. Regulations are thus established, and advice is provided on what is ‘acceptable’ exposure. Examples include work/rest cycles in the heat, hydration regimes, rates of ascent to and duration of stay at altitude and diving depth. While usually valuable and well intentioned, it is important to realise the breadth and importance of limitations associated with such guidelines. Regulations and advisories leave less room for self-determination, learning and perhaps adaptation. Regulations based on stress (e.g. work/rest cycles relative to WBGT) are more practical but less direct than those based on strain (e.g. core temperature), but even the latter can be substantively limited (e.g. by lack of criterion validation and allowance for behavioural regulation in the research on which they are based). *Extreme Physiology & Medicine* is publishing a series of reviews aimed at critically examining the issues involved with self- versus regulation-controlled human movement acutely and chronically in extreme environments. These papers, arising from a research symposium in 2013, are about the impact of people engaging in such environments and the effect of rules and guidelines on their safety, enjoyment, autonomy and productivity. The reviews will cover occupational heat stress, sporting heat stress, hydration, diving, extreme loading, chronic unloading and high altitude. Ramifications include factors such as health and safety, productivity, enjoyment and autonomy, acute and chronic protection and optimising adaptation.

## Main text

For a sub-tropical, air-breathing, low-altitude homeotherm, most of the planet Earth represents a hostile environment for humans and explains why they have populated only about 15% of the surface of the planet. Having said this, humans work, rest and play in immensely varied artificial and natural extreme environments, briefly or chronically, purposely or accidentally. Individuals’ behavioural and physiological capacity to endure and enjoy such environments also varies immensely, mainly between people but also in the same person at different times. The term ‘extreme’ typically refers to insufficiency or excess of one or more stressors, such as thermal energy, fluid availability, oxygen pressure, hydrostatic pressure, gravity or pollutants. The reactions associated with movement also cause stress of various types, which may potentiate or attenuate environmental stressors. The stress can be transiently adverse but chronically beneficial; for example, lack of mechanical and metabolic stress—as occurs in chronic inactivity or space travel—may be transiently safe but chronically harmful by virtue of insulin resistance, osteoporosis, sarcopenia, lack of skill and experience and so on. Individuals’ susceptibilities vary tremendously, due to factors such as genetics, acclimatisation, aerobic fitness, age and disease status.

Adverse effects of acute stress are readily identifiable, and attempts are made to regulate or advise against these threats, whereas chronic stress has less identifiable outcomes and is therefore less adequately reflected in regulations and advisory statements. For example, the cause of a fracture in healthy bone is usually clear, and so blame can be apportioned, but no one—other than perhaps the victim—is held culpable for a fracture caused by osteoporosis. Modern societies have increasingly sought to protect people from such stressors and, in that way, minimise their adverse effects. Regulations are established, and advice is provided on what is ‘acceptable’ exposure, leaving less room for self-determination, learning and perhaps adaptation. Examples include work/rest cycles in the heat, hydration regimes, rates of ascent to and duration of stay at altitude and diving depth. While usually valuable and well intentioned, it is important to realise the limitations associated with such guidelines. These include:

•Where should the bar be set and why. As alluded in the preceding paragraphs, even among the minority of regulations that use a measure of physiological strain (e.g. core temperature, heart rate, hypohydration, SaO_2_), it is just about impossible to determine a single value that is appropriate for all individuals in all circumstances.

•Are perceptions factored in, and if not, why not? In many cases, perceptions are the integral of an individual’s ability to cope with a stressor. However, they cannot always be relied upon.

•Regulations are often based on research aimed at elucidating acute and adaptive physiological responses to stress, using experimental designs that necessarily preclude acute or chronic behavioural regulation to some extent. Mechanistic experiments in humans typically also impose ethical end points (e.g. deep body temperature has to remain between 35°C–39.0°C) which further limit a thorough understanding of the acute or developmental role of experience and skill in tolerating and negotiating an extreme environment.

•Regulations introduce an expectation and accountability that can be problematic for both the regulator and the regulated. Like a speed limit to a motorist, a limit or end point can be regarded as a target rather than a guide among other indicators (of welfare).

•Regulations disempower individuals and allow the abrogation of person responsibility. They create the impression, rightly or wrongly, that personal experience and perceptions cannot be trusted. In some environments, this may be appropriate, but in some, it may impose unnecessary barriers, such that the cost of non-engagement is ultimately higher than of engagement. For example, living a sedentary existence in air-conditioned dwellings and workplaces provides no physiological or experiential resilience to the sustained heat exposure that would accompany a heat wave that overwhelmed an electricity grid or could not be countered technologically for financial reasons.

Perhaps one inadvertent outcome of regulations and advisory statements are that they highlight the dangers of overtly extreme environments and thus help propagate public discourse that emphasises the legitimacy of acute protection and the status quo. The risk of dying by climbing to the highest point on earth is about one fifth that of dying early due to living a sedentary and thus supposedly safe life (approximately 2% vs. 10%). Yet one’s living space—which is what promotes sedentariness—is regarded as being immeasurably safer than Mt. Everest. Admittedly, the duration of exposure is vastly greater in the case of being sedentary but so is the population-attributable risk.

A central consideration is whether humans are equipped with the afferent, integrative and efferent processes to adequately transduce and respond to relevant stressors. That is, are our perceptions of thirst, body tissue temperatures, hypoxia, etc., sufficient and adequate in all circumstances? Even for these relatively simple stimuli, expert opinions and recommendations can be polarised (e.g. [1-4]). Some stimuli are clearly dangerous by virtue of their nature; they can incapacitate due to their sudden or insidious onset, lack of receptors for detection or their direct effect on the central nervous system—especially at the extremes of pressure (high altitude and diving). Such stimuli include altitude-mediated reductions in oxygen pressure causing severe pulmonary or cerebral oedema and nitrogen emboli with decompression. Similarly, insidious hypothermia can develop under cold protection ensembles due to rate of skin cooling being too slow to trigger rate-sensitive thermoafferent drive—an effect that is absent for heat stress. Notably, most dangers occur largely during exposures arising from artificially-assisted or protected and/or unfamiliar circumstances (except where rapid ascents and descents can occur naturally, such as climbing Mt. Kilimanjaro or weight-assisted free diving, respectively).

Another key consideration is whether humans can adapt successfully to a given environment. If they can, how is this optimised, how much is behavioural and how much physiological, is it biphasic (i.e. initially increasing the power of a physiological response but then giving way to improved cellular efficiency and tissue structure [5,6]), can everyone adapt, how quickly can people adapt and what are the best markers of adaptation, does adaptation to one environmental stressor provide cross tolerance to another, is it ergogenic, and so on. For many extreme environments, these questions have not been satisfactorily answered.

*Extreme Physiology and Medicine* is publishing a series of reviews aimed at critically examining the issues involved with self- versus regulation-controlled human movement acutely and chronically in extreme environments. These papers are about the impact of people engaging in such environments and the effect of rules and guidelines on their safety, enjoyment, autonomy and productivity. The following questions are addressed, having formed the basis of a symposium hosted by the School of Physical Education, Sport and Exercise Sciences, University of Otago, in February 2013: (a) What are the stressors or dangers, for human movement? (b) What regulations are established, and why/how are they set? (c) Pros and cons of self versus prescribed acute exposure; (d) can people adapt and is this desirable (i.e. adaptations or maladaptations)? (e) Pros and cons of self versus prescribed chronic/adaptive exposure; and (f) suggestions and future directions for practice and research. The reviews will cover occupational heat stress, sporting heat stress, hydration, diving, extreme loading, chronic unloading and high altitude. Ramifications include factors such as health and safety, productivity, enjoyment and autonomy, acute and chronic protection and optimising adaptation. Flow on effects, which are outside the scope of the current debate, but nevertheless important, include diverse issues such as insurance requirements and policies, personal responsibility, liability and freedom, the bottled water industry, liability and resourcing for hosting recreational activities.

## Abbreviations

SaO2: saturation of oxygen in arterial blood; WBGT: wet bulb globe temperature.

## Competing interests

The authors declare that they have no competing interests.

## Authors’ contributions

JC and MT both wrote and approved the manuscript.
